# Exposure–Efficacy Analyses Support Optimal Dosing Regimens of Ceftolozane/Tazobactam in Participants with Hospital-Acquired/Ventilator-Associated Bacterial Pneumonia in ASPECT-NP

**DOI:** 10.1128/aac.01399-21

**Published:** 2022-04-26

**Authors:** Wei Gao, Julie Passarell, Yogesh T. Patel, Zufei Zhang, Gina Lin, Jill Fiedler-Kelly, Christopher J. Bruno, Elizabeth G. Rhee, Carisa S. De Anda, Hwa-Ping Feng

**Affiliations:** a Merck & Co., Inc., Kenilworth, New Jersey, USA; b Cognigen Corporation, a Simulations Plus Company, Buffalo, New York, USA

**Keywords:** ceftolozane, exposure–efficacy, hospital-acquired bacterial pneumonia, tazobactam, ventilator-associated bacterial pneumonia

## Abstract

An exposure–efficacy analysis of the phase 3 ASPECT-NP trial was performed to evaluate the relationship between plasma exposure of ceftolozane and tazobactam and efficacy endpoints (primary: 28-day all-cause mortality; key secondary: clinical cure at test-of-cure visit) in adult participants with hospital-acquired or ventilator-associated bacterial pneumonia (HABP/VABP). Participants (*N *=* *231) from the ceftolozane/tazobactam treatment group in the intention-to-treat population who had pharmacokinetic data available and relevant baseline lower respiratory tract (LRT) pathogen(s) susceptibility data were included. Population pharmacokinetic models were used to predict individual ceftolozane and tazobactam plasma exposure measures (percentage of the interdose interval with free drug concentrations above the MIC [%*ƒ*T>MIC] and %*ƒ*T above a threshold [%*ƒ*T>C_T_ = 1 μg/mL], respectively) associated with the last dose using the highest ceftolozane/tazobactam MIC for the relevant baseline LRT pathogens. Efficacy measures were comparable between the baseline LRT pathogens and across MIC cutoffs (1–8 μg/mL). Most participants (82%) had 99% *ƒ*T>MIC for ceftolozane; 9% (*N *=* *21/231) had 0% ƒT>MIC due to high MICs of the LRT pathogen (64–256 μg/mL). The %ƒT>MIC for ceftolozane exceeded 73% for all participants with baseline LRT pathogen(s) MIC ≤4 μg/mL. All 231 participants achieved the tazobactam pharmacokinetic/pharmacodynamic target of >20% *ƒ*T>C_T_ where C_T_ = 1 μg/mL. For either efficacy endpoint, median ceftolozane %ƒT>MIC was 99% in participants achieving efficacy. No exposure–efficacy trend was observed for ceftolozane or tazobactam. These results further support the recommended ceftolozane/tazobactam dosing regimens evaluated in ASPECT-NP for patients with HABP/VABP.

## INTRODUCTION

Nosocomial pneumonia, comprising hospital-acquired bacterial pneumonia (HABP) and ventilator-associated bacterial pneumonia (VABP), is the most common health care–associated infection in the United States (1). All-cause mortality ranges associated with nosocomial pneumonia are 10% to 22% for patients with nonventilated HABP, 10% to 27% for patients with VABP, and 15% to 39% for patients with ventilated HABP (2). Pseudomonas aeruginosa and *Enterobacterales*—the predominant Gram-negative causative pathogens of HABP/VABP—are often nonsusceptible to commonly used antibacterial agents (3–5). Novel antibacterial therapies with broad activity against resistant causative pathogens may not only reduce mortality among patients with HABP/VABP but also the burden on health care resources associated with the treatment of these infections (6–9).

Ceftolozane/tazobactam, an intravenous combination antibacterial agent composed of the antipseudomonal cephalosporin ceftolozane and the established β-lactamase inhibitor tazobactam, is approved for the treatment of adults with complicated intra-abdominal infections (used in combination with metronidazole), complicated urinary tract infections, including pyelonephritis, and HABP/VABP (10, 11). *In vitro*, ceftolozane/tazobactam was shown to potently inhibit *Enterobacterales* and P. aeruginosa isolates obtained from patients in the United States and Europe with various infection types, including HABP/VABP (12–14). A 3-g dose of ceftolozane/tazobactam (2 g of ceftolozane and 1 g of tazobactam), with dosing adjusted based on renal function, was evaluated in participants with ventilated HABP and VABP in the phase 3, randomized, controlled, double-blind ASPECT-NP trial (10, 15). Among participants who received ceftolozane/tazobactam, 28-day all-cause mortality (primary endpoint) and clinical cure rates at the test-of-cure (TOC) visit (key secondary endpoint) were found to be noninferior to participants who received 1 g of meropenem; ceftolozane/tazobactam was also well tolerated and had similar rates of adverse events (AEs) compared with meropenem (15).

Population pharmacokinetic (PK) models developed based on data from multiple clinical studies, including the ASPECT-NP trial, were used to predict ceftolozane and tazobactam PK exposures in the plasma and pulmonary epithelial lining fluid (16, 17). Two-compartment models with zero-order input and first-order elimination best described the plasma concentration–time profiles of both ceftolozane and tazobactam (16). As expected for renally eliminated drugs (18), renal function as measured by creatinine clearance (CrCl) was a significant covariate on both ceftolozane and tazobactam clearance. The potential relationships between ceftolozane and tazobactam exposures, resulting from the 3-g (or renally adjusted) ceftolozane/tazobactam dose, and response among patients with HABP/VABP have not been previously evaluated. The objective of this ASPECT-NP data analysis was to characterize the relationship between efficacy endpoints and plasma exposures for ceftolozane and tazobactam, estimated using the above-mentioned population PK models, in adult participants with ventilated HABP/VABP.

## RESULTS

### Data description and participant baseline characteristics.

The source data set for the exposure–efficacy analyses (EEA population) included the 361 participants who received ceftolozane/tazobactam in the intention-to-treat (ITT) population. Of these 361 participants, 130 were excluded from this analysis owing to not having exposure measures for the following reasons: (1) no relevant lower respiratory tract (LRT) pathogens were identified and thus no susceptibility information was available; (2) all identified baseline LRT pathogens were resistant to both study drugs (ceftolozane/tazobactam and meropenem), as these resistant LRT pathogens were excluded from the analysis; and (3) no PK data were available. Ceftolozane and tazobactam exposures of the remaining 231 participants were included in the EEA population data set for both efficacy endpoints.

Participants included in the EEA data set were primarily white (84.0%), male (75.3%), and had a mean age of 60 years, with 43.3% of participants aged ≥65 years (Table 1). Covariates that impacted PK (renal function as measured by CrCl, pneumonia, and body weight), in addition to race, sex, and age, are provided in Table S1. Participants were divided by baseline renal function as estimated by the local laboratory into the following subgroups: CrCl ≥15 to 29 mL/min (*N *=* *9; 3.9%); CrCl ≥30 to ≤50 mL/min (*N *=* *16; 6.9%); CrCl >50 mL/min (*N *=* *206; 89.2%; Table 2). Mild, moderate, or severe renal impairment, as assessed by baseline CrCl from central laboratory data, was reported in 32.9% of participants (Table S1). Participant baseline characteristics in this subset were comparable to those in the ITT population in the ceftolozane/tazobactam group (Table S1).

**TABLE 1:** Participant baseline characteristics in the EEA population^*a*^

Characteristic	Participants (*N *=* *231)
Age, yrs, mean (SD)	60.0 (16.4)
Age group, *n* (%)	
<65 yrs	131 (56.7)
≥65 yrs	100 (43.3)
≥75 yrs	47 (20.3)
Male sex, *n* (%)	174 (75.3)
Baseline body wt, kg, mean (SD)	81.2 (16.6)
Baseline CrCl, mL/min, mean (SD)	108.5 (60.0)

a CrCl, creatinine clearance; EEA, exposure–efficacy analysis; LRT, lower respiratory tract.

**TABLE 2:** Number of participants included in the exposure–efficacy analyses by baseline renal function subgroup, with corresponding ceftolozane/tazobactam dose adjustments^*a*^

Renal function subgroup	Ceftolozane, mg	Tazobactam, mg	Participants, *n* (%) (*N *=* *231)
CrCl ≥15 to <30 mL/min	500	250	11 (4.8)
CrCl ≥30 to ≤50 mL/min	1000	500	19 (8.2)
CrCl >50 to <80 mL/min	2000	1000	46 (19.9)
CrCl ≥80 to <150 mL/min	2000	1000	109 (47.2)
CrCl ≥150 mL/min	2000	1000	46 (19.9)

a CrCl, creatinine clearance. Doses were determined based on renal function as estimated by CrCl, which was assessed at the local laboratory. The dose for some participants was adjusted during treatment to account for changes in CrCl.

### Overall efficacy.

The participants included in the EEA population had an overall 28-day all-cause mortality rate of 16% (*N *=* *36/231) and a TOC clinical cure rate of 65% (*N *=* *151/231). Efficacy outcomes were comparable between participants with *Enterobacterales* and P. aeruginosa in baseline LRT cultures, with 28-day all-cause mortality rates of 13.0% and 23.3%, respectively, and clinical cure rates at TOC of 67.5% and 65.1%, respectively (Table 3). Rates of PK/pharmacodynamic (PD) target attainment for ceftolozane and tazobactam were also comparable for participants with baseline *Enterobacterales* and P. aeruginosa pathogens (Table 3). When primary and key secondary efficacy outcomes were evaluated by baseline LRT pathogen MIC values for ceftolozane/tazobactam, 28-day all-cause mortality rates ranged from 6.3% to 25.6% and clinical cure rates at TOC from 27.3% to 75.0% (Table 4). Sample sizes were small in the baseline MIC subgroups of >1 to ≤2 μg/mL, >2 to ≤4 μg/mL, and >4 to ≤8 μg/mL. Baseline MIC subgroups of ≤1 μg/mL and >8 to ≤256 μg/mL had larger sample sizes, and the 28-day all-cause mortality rate was higher and clinical cure rate was relatively lower in the baseline MIC >8- to ≤256-μg/mL subgroups, consistent with the impact of baseline MIC values on the PK/PD index of ceftolozane.

**TABLE 3:** Summary statistics of exposure measurements and primary and key secondary efficacy outcomes by the baseline pathogen^*a*^

Variable	Statistic	*Enterobacterales*^*b*^ (*n *=* *154)	Pseudomonas aeruginosa^*b*^ (*n *=* *43)	Overall^*b*^ (*N *=* *191)
%*ƒ*T>C_T_ >1 μg/mL for tazobactam	Mean (SD)	76.1 (24.6)	79.7 (23.1)	76.9 (24.3)
	Median (min–max)	87.2 (28.0–99.0)	94.4 (22.8–99.0)	87.2 (22.8–99.0)
%*ƒ*T>MIC for ceftolozane	Mean (SD)	84.3 (32.8)	94.8 (16.1)	86.6 (30.3)
	Median (min–max)	99.0 (0.0–99.0)	99.0 (0.0–99.0)	99.0 (0.0–99.0)
Ceftolozane/tazobactam MIC, μg/mL	Mean (SD)	23.1 (58.8)	8.8 (39.2)	20.0 (55.4)
	Median (min–max)	0.5 (0.1–256.0)	1.0 (0.3–256.0)	0.5 (0.1–256.0)
28-day all-cause mortality,^*c*^ *n* (%)	Death	20 (13.0)	10 (23.3)	30 (15.2)
Clinical cure at TOC,^*d*^ *n* (%)	Cure	104 (67.5)	28 (65.1)	132 (67.0)

a %*f*T>C_T_ >1 μg/mL, percentage of time the threshold concentration of tazobactam exceeded 1 μg/mL; %*f*T>MIC, percentage of time the concentration of free ceftolozane in plasma exceeded the MIC that was determined in the presence of tazobactam; EOT, end of treatment; max, maximum; min, minimum; TOC, test of cure.

b The analysis set presented in this table is a subset of the exposure–efficacy analysis set. Six participants had two baseline pathogens with the same highest baseline MIC value and were included in both the *Enterobacterales* and P. aeruginosa groups.

c Mortality was assessed on days 14 and 28; the 28-day all-cause mortality endpoint accounted for deaths on or before day 28.

d Clinical response assessments were performed at the EOT, TOC, and late follow-up visits. Determination of clinical response was based on an overall assessment of clinical status based on signs, symptoms, and available laboratory data. Clinical responses at EOT and TOC visits were classified as cure, failure, or indeterminate. “Clinical cure” was a favorable clinical response.

**TABLE 4:** Summary of primary and key secondary efficacy endpoints by baseline MIC cutoff values for ceftolozane/tazobactam^*a*^

MIC, μg/mL	28-day all-cause mortality, *n*/*N* (%)	Clinical cure at TOC, *n*/*N* (%)
≤1	19/142 (13.4)	102/142 (71.8)
>1 to ≤2	3/19 (15.8)	11/19 (57.9)
>2 to ≤4	1/16 (6.3)	12/16 (75.0)
>4 to ≤8	2/11 (18.2)	3/11 (27.3)
>8 to ≤256	11/43 (25.6)	23/43 (53.5)

a TOC, test of cure. Due to the small number of isolates at MIC cutoff values of >8 to ≤256 μg/mL, participants within this range were combined for analysis. The MIC value used in this analysis corresponded to the highest MIC for relevant pathogens identified at baseline for each participant.

### Ceftolozane.

**FIG 1:**
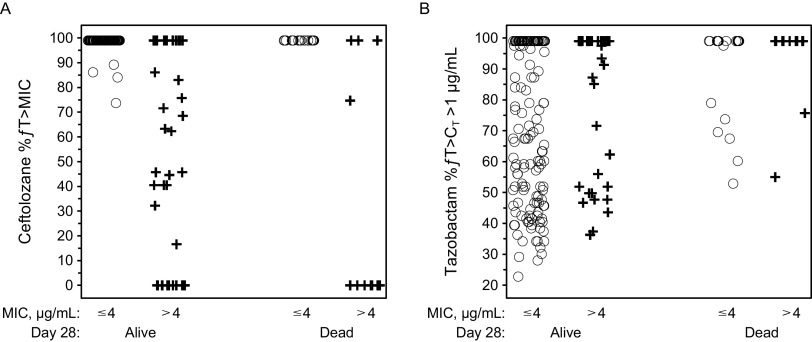
Scatterplots of 28-day all-cause mortality stratified by baseline MIC category (≤4, >4 μg/mL) for (A) ceftolozane (%*ƒ*T>MIC) and (B) tazobactam (%*ƒ>*C_T_ >1 μg/mL). %*ƒ*T>MIC, percentage of time the concentration of free ceftolozane in plasma exceeded the MIC; %*ƒ>*C_T_ >1 μg/mL, percentage of time the concentration of free tazobactam in plasma exceeded the threshold concentration of 1 μg/mL.

The exposure–efficacy data for ceftolozane were bimodal in distribution. Overall, 82% of participants (*N *=* *190/231) had %*ƒ*T>MIC of 99%. Among the remaining participants, 9% (*N *=* *21/231) had 0% *ƒ*T>MIC due to high MIC values of their baseline LRT isolates (64–256 μg/mL), which were notably higher than the current European Committee on Antimicrobial Susceptibility Testing breakpoint of ≤4 μg/mL for P. aeruginosa (19). Exploratory graphical analyses of ceftolozane exposure measures by 28-day all-cause mortality stratified by MIC ≤4 μg/mL and MIC >4 μg/mL indicated that ceftolozane %*ƒ*T>MIC values were comparable between participants who had died and those who survived at day 28 (Fig. 1A).

**FIG 2:**
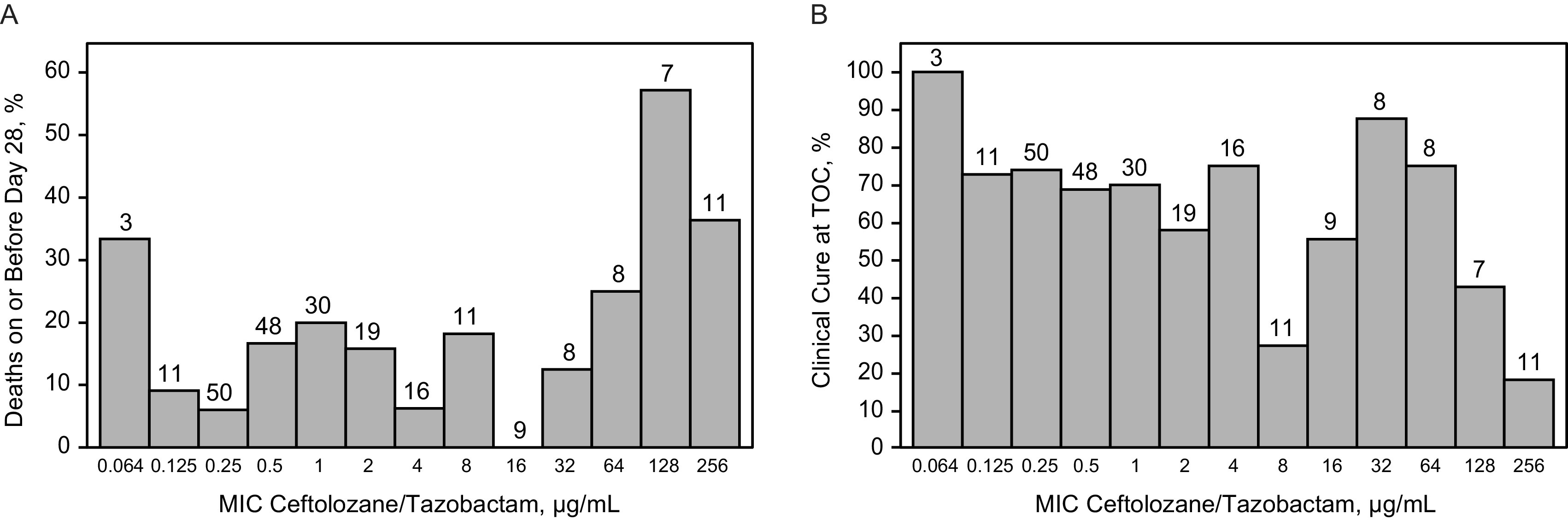
Frequency distributions of baseline MIC category by (A) 28-day all-cause mortality and (B) clinical cure at TOC. The number above each bar represents the number of participants in each baseline MIC category. TOC, test of cure.

**FIG 3:**
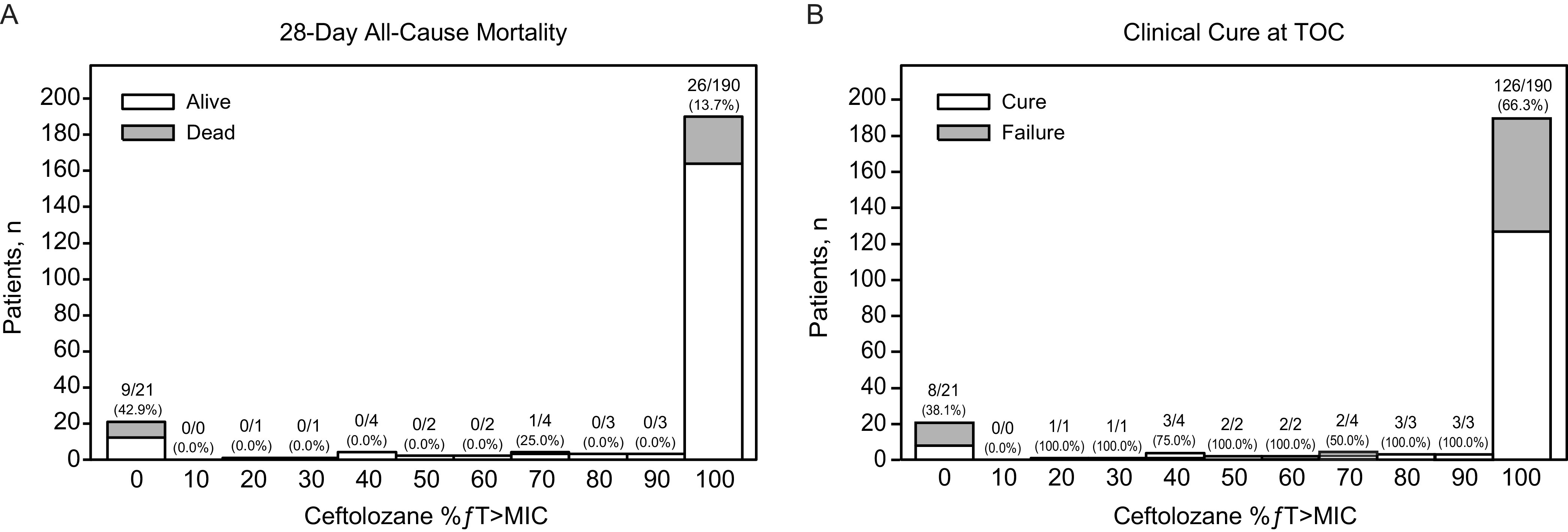
Frequency distributions of exposure measures for ceftolozane (%*ƒ*T>MIC) by (A) 28-day all-cause mortality and by (B) clinical cure at TOC. The number under each bar along the *x* axis represents the median of the range of values for that bar. The ratio of numbers represents the number of deaths/total number of participants in each bar. The number in parentheses is the percentage of participants who died. %*ƒ*T>MIC, percentage of time the concentration of free ceftolozane in plasma exceeded the MIC; TOC, test of cure.

Outcomes by ceftolozane exposure were analyzed in participants based on the MIC of their baseline LRT pathogens for ceftolozane/tazobactam treatment (Table 5; Fig. 2). Baseline MIC cutoff values were ≤1, ≤2, ≤4, and ≤8 μg/mL (Table 5). Overall, 177 participants had a baseline LRT pathogen at or below the 4-μg/mL MIC cutoff (susceptibility breakpoint for P. aeruginosa); of these participants, 173 had 99% *ƒ*T>MIC and 4 had <99% *ƒ*T>MIC. The all-cause mortality rate for participants with 99% *ƒ*T>MIC for ceftolozane was 13.3% (*N *=* *23/173) compared with 0.0% (*N *=* *4/173) for the participants with <99% *ƒ*T>MIC. No exposure–efficacy trend for 28-day all-cause mortality was observed among participants with a pathogen with a MIC at or below the 4-μg/mL susceptibility breakpoint for P. aeruginosa (Fig. 2A), and most participants had 99% *ƒ*T>MIC (Fig. 3A). The exploratory data analyses found no trend; therefore, the data did not support meaningful exploration of models, and formal exposure–efficacy modeling was not conducted for the 28-day all-cause mortality endpoint.

**TABLE 5:** Summary of primary and key secondary efficacy endpoints by %*f*T>MIC and MIC cutoff values for ceftolozane/tazobactam^*a*^

MIC cutoff, μg/mL	Exposure	*n*	%*ƒ*T>MIC^*b*^	28-day all-cause mortality, %	Clinical cure at TOC, %
≤1	%*f*T>MIC of 99.0%	141	NA	13.5	71.6
	%*f*T>MIC <99.0%	1	86.1%	0.0	100.0
≤2	%*f*T>MIC of 99.0%	160	NA	13.8	70.0
	%*f*T>MIC <99.0%	1	86.1%	0.0	100.0
≤4	%*f*T>MIC of 99.0%	173	NA	13.3	70.0
	%*f*T>MIC <99.0%	4	73.7%, 84.0%, 86.1%, 89.2%	0.0	100.0
≤8	%*f*T>MIC of 99.0%	182	NA	13.7	67.6
	%*f*T>MIC <99.0%	6	63.3%, 68.5%, 73.7%, 84.0%, 86.1%, 89.2%	0.0	83.3

a %*f*T>MIC, percentage of time the concentration of free ceftolozane in plasma exceeded the MIC; NA, not applicable; TOC, test of cure.

b Individual values for each pathogen with <99% *f*T>MIC are listed individually.

Ceftolozane %*ƒ*T>MIC values were also comparable between participants who did and did not achieve clinical cure at TOC, with median values of 99% in both groups (Fig. 3B). No exposure–efficacy trend was observed for clinical cure rates at TOC for any of the baseline MIC cutoffs (Fig. 2B); at the 4-μg/mL cutoff, the clinical cure rate for participants with 99% *ƒ*T>MIC was approximately 70% compared with 100% for participants with <99% *ƒ*T>MIC (Table 5). Therefore, as with 28-day all-cause mortality, formal exposure–efficacy modeling was not feasible for clinical cure rates at TOC.

### Tazobactam.

**FIG 4:**
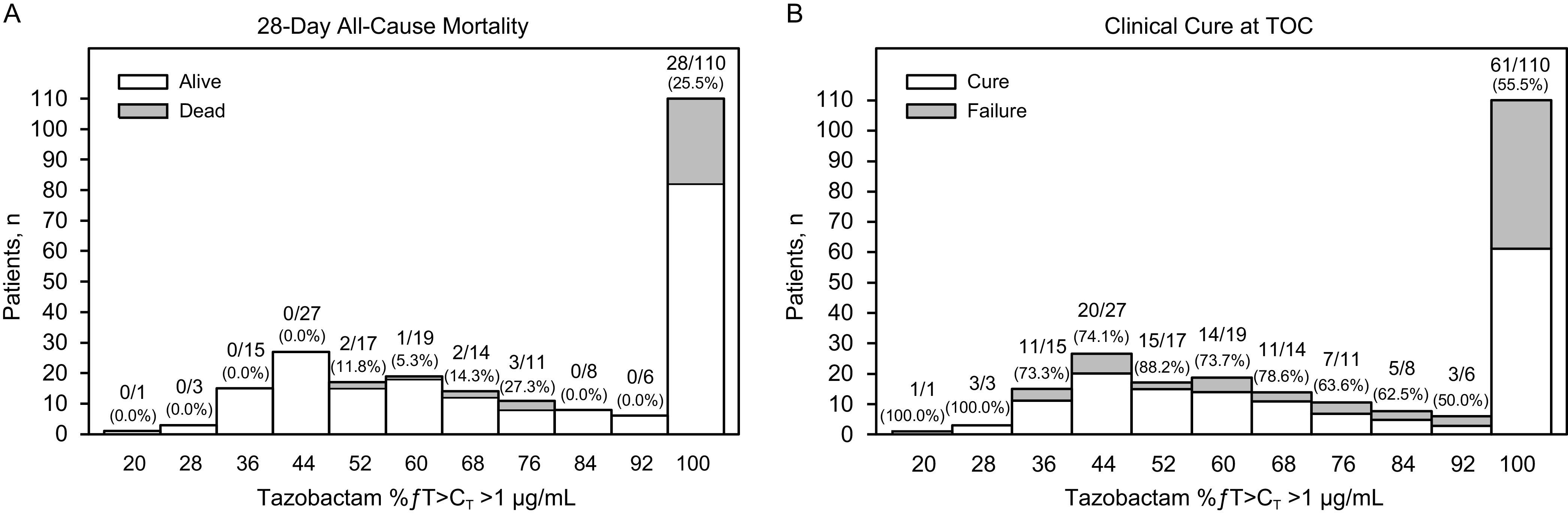
Frequency distributions of exposure measures for tazobactam (%*ƒ*T>C_T_ >1 μg/mL) by (A) 28-day all-cause mortality and by (B) clinical cure at TOC. The number under each bar along the *x* axis represents the median of the range of values for that bar. The ratio of numbers represents the number of deaths/total number of participants in each bar. The number in parentheses is the percentage of participants who died. %*ƒ*T>C_T_ >1 μg/mL, percentage of time the concentration of free tazobactam in plasma exceeded the threshold concentration of 1 μg/mL; TOC, test of cure.

All participants in the EEA population achieved the tazobactam PK/PD target of 20% *ƒ*T>C_T_, where C_T_=1 μg/mL. An exploratory graphical analysis of tazobactam exposures (%*ƒ*T>C_T_ >1 μg/mL) by 28-day all-cause mortality stratified by ceftolozane MIC ≤4 μg/mL and MIC >4 μg/mL indicated tazobactam exposures were higher in patients who died compared with those who survived (Fig. 1B). In addition, rates of 28-day all-cause mortality increased with increasing tazobactam %*ƒ*T>C_T_ (Fig. 4A). Clinical cure rates decreased with increasing %*ƒ*T>C_T_, with a mean %*ƒ*T>C_T_ >1 μg/mL of 73.0% in participants who achieved clinical cure compared with 84.5% in those who experienced clinical failure at TOC (Fig. 4B). Tazobactam has no intrinsic antibacterial activity; rather, tazobactam inhibits several class A β-lactamases, thereby restoring the antibacterial activity of ceftolozane for organisms expressing these enzymes (20–22). The trend of decreasing efficacy with increasing tazobactam PK/PD target values suggests a lack of a direct, causal relationship between tazobactam PK and the efficacy endpoints. Therefore, no formal exposure–efficacy modeling was conducted for all-cause mortality or clinical response with tazobactam exposure measures.

Efficacy outcomes were further analyzed based on tazobactam exposure in participants whose baseline LRT isolate was at or below various ceftolozane/tazobactam MIC values (1, 2, 4, and 8 μg/mL; Table 6), and no exposure–efficacy trend was found at any MIC cutoff. Outcomes by renal function group are summarized in Table S2. Compared with participants with mild renal impairment/normal renal function (CrCl >50 mL/min), there was a trend toward higher rates of 28-day all-cause mortality and lower rates of clinical response in participants with moderate/severe renal impairment (CrCl ≤50 mL/min).

**TABLE 6:** Summary of pharmacokinetics and primary and key secondary efficacy endpoints by baseline MIC cutoff values for ceftolozane/tazobactam^*a*^

Variable	Statistic	MIC ≤1 μg/mL (*n *=* *142)	MIC >1 μg/mL (*n *=* *89)	Overall (*N *=* *231)
%*ƒ*T>C_T_ >1 μg/mL for tazobactam	Mean (SD)	72.6 (24.8)	83.9 (22.1)	77.0 (24.4)
	Median (min–max)	74.7 (22.8–99.0)	99.0 (36.3–99.0)	89.2 (22.8–99.0)
%*ƒ*T>MIC for ceftolozane	Mean (SD)	98.9 (1.1)	67.3 (41.7)	86.7 (30.1)
	Median (min–max)	99.0 (86.1–99.0)	99.0 (0.0–99.0)	99.0 (0.0–99.0)
Ceftolozane/tazobactam MIC, μg/mL	Mean (SD)	0.5 (0.3)	54.1 (83.9)	21.1 (58.1)
	Median (min–max)	0.5 (0.1–1.0)	8.0 (2.0–256.0)	1.0 (0.1–256.0)
28-day all-cause mortality,^*b*^ *n* (%)	Death	19 (13.4)	17 (19.1)	36 (15.6)
Clinical cure at TOC visit,^*c*^ *n* (%)	Cure	102 (71.8)	49 (55.1)	151 (65.4)

Variable	Statistic	**MIC ≤2 μg/mL** (*n* =* *161)	**MIC >2 μg/mL** (*n *=* *70)	**Overall** (*N *=* *231)
%*ƒ*T>C_T_ >1 μg/mL for tazobactam	Mean (SD)	73.5 (24.8)	84.8 (21.6)	77.0 (24.4)
	Median (min–max)	76.8 (22.8–99.0)	99.0 (36.3–99.0)	89.2 (22.8–99.0)
%*ƒ*T>MIC for ceftolozane	Mean (SD)	98.9 (1.0)	58.7 (43.2)	86.7 (30.1)
	Median (min–max)	99.0 (86.1–99.0)	75.2 (0.0–99.0)	99.0 (0.0–99.0)
Ceftolozane/tazobactam MIC, μg/mL	Mean (SD)	0.7 (0.6)	68.2 (89.6)	21.1 (58.1)
	Median (min–max)	0.5 (0.1–2.0)	16.0 (4.0–256.0)	1.0 (0.1–256.0)
28-day all-cause mortality,^*b*^ *n* (%)	Death	22 (13.7)	14 (20.0)	36 (15.6)
Clinical cure at TOC visit,^*c*^ *n* (%)	Cure	113 (70.2)	38 (54.3)	151 (65.4)

Variable	Statistic	**MIC ≤4 μg/mL** (*n* =* *177)	**MIC >4 μg/mL** (*n *=* *54)	**Overall** (*N *=* *231)
%*ƒ*T>C_T_ >1 μg/mL for tazobactam	Mean (SD)	74.4 (24.7)	85.3 (21.6)	77.0 (24.4)
	Median (min–max)	78.9 (22.8–99.0)	99.0 (36.3–99.0)	89.2 (22.8–99.0)
%*ƒ*T>MIC for ceftolozane	Mean (SD)	98.6 (2.5)	47.7 (43.3)	86.7 (30.1)
	Median (min–max)	99.0 (73.7–99.0)	45.2 (0.0–99.0)	99.0 (0.0–99.0)
Ceftolozane/tazobactam MIC, μg/mL	Mean (SD)	1.0 (1.1)	87.3 (94.0)	21.1 (58.1)
	Median (min–max)	0.5 (0.1–4.0)	32.0 (8.0–256.0)	1.0 (0.1–256.0)
28-day all-cause mortality,^*b*^ *n* (%)	Death	23 (13.0)	13 (24.1)	36 (15.6)
Clinical cure at TOC visit,^*c*^ *n* (%)	Cure	125 (70.6)	26 (48.1)	151 (65.4)

Variable	Statistic	**MIC ≤8 μg/mL** (*n *=* *188)	**MIC >8 μg/mL** (*n *=* *43)	**Overall** (*N *=* *231)
%*ƒ*T>C_T_ >1 μg/mL for tazobactam	Mean (SD)	75.2 (24.8)	84.7 (21.3)	77.0 (24.4)
	Median (min–max)	81.0 (22.8–99.0)	99.0 (37.4–99.0)	89.2 (22.8–99.0)
%*ƒ*T>MIC for ceftolozane	Mean (SD)	98.3 (4.2)	36.1 (40.7)	86.7 (30.1)
	Median (min–max)	99.0 (63.3–99.0)	16.6 (0.0–99.0)	99.0 (0.0–99.0)
Ceftolozane/tazobactam MIC, μg/mL	Mean (SD)	1.4 (2.0)	107.5 (95.3)	21.1 (58.1)
	Median (min–max)	0.5 (0.1–8.0)	64.0 (16.0–256.0)	1.0 (0.1–256.0)
28-day all-cause mortality,^*b*^ *n* (%)	Death	25 (13.3)	11 (25.6)	36 (15.6)
Clinical cure at TOC visit,^*c*^ *n* (%)	Cure	128 (68.1)	23 (53.5)	151 (65.4)

a %*f*T>C_T_ >1 μg/mL, percentage of time the concentration of free tazobactam in plasma exceeded the threshold concentration of 1 μg/mL; %*f*T>MIC, percentage of time the concentration of free ceftolozane in plasma exceeded the MIC; EOT, end of treatment; max, maximum; min, minimum; TOC, test of cure.

b Mortality was assessed on days 14 and 28; the 28-day all-cause mortality endpoint accounted for deaths on or before day 28.

c Clinical response assessments were performed at the EOT, TOC, and late follow-up visits. Determination of clinical response was based on an overall assessment of clinical status based on signs, symptoms, and available laboratory data. Clinical responses at EOT and TOC visits were classified as cure, failure, or indeterminate. “Clinical cure” was a favorable clinical response.

## DISCUSSION

This study evaluated potential relationships between ceftolozane and tazobactam exposures measured via established PK/PD indices (23, 24) and efficacy outcomes, including 28-day mortality rates in the phase 3 ASPECT-NP trial (15). As %*ƒ*T>MIC is the ceftolozane PK/PD parameter that is most predictive of *in vitro* efficacy, in the current analysis, we explored the potential exposure–efficacy relationship based on various MIC categories of baseline infecting pathogens.

No consistent exposure–efficacy trends were identified for ceftolozane, except at the highest MIC values where an increase in 28-day all-cause mortality and a decrease in clinical cure at TOC were detected, or for tazobactam in either primary or key secondary efficacy endpoints. All participants with a baseline LRT pathogen with an MIC ≤4 μg/mL (i.e., the highest susceptibility breakpoint) achieved exposures above the PK/PD targets for both components of ceftolozane/tazobactam.

No interpretable trends between the efficacy endpoints and tazobactam %*ƒ*T> C_T_ (1 μg/mL) were identified. Of the 231 participants in the EEA data set, the relevant pathogens from 177 participants had a highest baseline ceftolozane/tazobactam MIC of ≤4 μg/mL. The highest proposed susceptibility breakpoint is 4 μg/mL for ceftolozane/tazobactam; therefore, this summary focuses on these participants. The ceftolozane plasma %*ƒ*T>MIC for all 177 participants was >30%. Notably, the ceftolozane %*ƒ*T>MIC distribution was highly skewed; ceftolozane plasma %*ƒ*T>MIC for 173 of the 177 participants was 99%, and the lowest %*ƒ*T>MIC in the remaining 4 participants was 73.7%. The all-cause mortality and clinical cure rates in these 177 participants were 13.3% and 70%, respectively. For the 4 participants with ceftolozane %*ƒ*T>MIC <99%, the all-cause mortality rate was 0% and the clinical cure rate was 100%. Together, these observations suggest a lack of an association between ceftolozane exposure and the efficacy endpoints. In the exposure–efficacy data set, there were differences in the all-cause mortality and clinical response rates between participants with moderate/severe renal impairment (CrCl ≤50 mL/min) and those with mild renal impairment or normal renal function (CrCl >50 mL/min). These results are consistent with a previous secondary analysis of ASPECT-NP that assessed the safety and efficacy of ceftolozane/tazobactam in participants with renal impairment (25). In that analysis, rates of 28-day all-cause mortality were higher in those with renal impairment compared with those with normal renal function, for both the ceftolozane/tazobactam and the meropenem treatment groups (25). These results likely reflect the established independent association between renal insufficiency and increased risk of mortality (26, 27). Huntington et al. (25) also reported that clinical cure rates were lower among participants with moderate/severe renal impairment compared with those with normal renal function for both the ceftolozane/tazobactam and the meropenem treatment groups. Another previously reported secondary analysis of ASPECT-NP participant data examined the relationship between augmented renal function, ceftolozane and tazobactam exposure levels, and clinical outcomes (28). Exposure decreased in both plasma and pulmonary epithelial lining fluid as CrCl increased from ≥80 mL/min to <150 mL/min to ≥210 mL/min; however, rates of 28-day all-cause mortality, clinical cure, and per-participant microbiologic cure were comparable between participants with normal renal function and those with augmented renal clearance who were treated with ceftolozane/tazobactam (28). Probability of target attainment was high for ceftolozane and tazobactam, regardless of renal function status (28). These results, coupled with the high efficacy in the MIC ≤4-μg/mL subgroup, provide support for the dosing regimens evaluated in ASPECT-NP as appropriate for the treatment of HABP/VABP at MIC values up to the P. aeruginosa susceptibility breakpoint of 4 μg/mL. These results are similar to the findings of an exploratory exposure–response analyses of 903 participants who received ceftazidime-avibactam in which no meaningful exposure–response relationships were found; nearly all participants achieved close to 100% for both ceftazidime %*ƒ*T>MIC and avibactam %*ƒ*T>C_T_ (29).

At the highest susceptibility breakpoint of 4 μg/mL for P. aeruginosa, the percentages of participants at day 28 that died and the percentage of participants who achieved clinical cure were similar to those participants with baseline pathogen MICs of >4 μg/mL. The highest MIC values for the relevant baseline pathogens identified for each participant were used in the analysis. A wide data distribution around the MIC was observed; of 231 participants, 142 had baseline pathogens with a maximum MIC of ≤1 μg/mL, 43 participants had baseline pathogens with a maximum MIC of >8 to ≤256 μg/mL, and relatively few participants had baseline pathogens with MIC values >1 to ≤8 μg/mL. The uneven sample sizes in these groups preclude definitive conclusions regarding exposure–efficacy trends for participants who had baseline pathogens with MIC values >8 μg/mL.

An exposure–safety analysis was planned to include AEs that met the following criteria: an observed incidence rate of >5% and an incidence rate in the ceftolozane/tazobactam treatment group that was 1.5-fold greater than that observed in the meropenem group. However, no AEs met the predefined criteria to be included in the exposure–safety analysis (data not shown), and thus no formal modeling analysis was conducted to evaluate the relationship between ceftolozane or tazobactam PK and safety.

In conclusion, this analysis indicates that there was no apparent correlation between the individual ceftolozane or tazobactam PK/PD index values and primary and key secondary efficacy endpoints in ASPECT-NP. These results suggest that adequate exposure was achieved at the doses evaluated in ASPECT-NP, and therefore provide additional support for the appropriateness of the recommended dosing regimen of ceftolozane/tazobactam 3 g (ceftolozane 2 g/tazobactam 1 g), adjusted based on renal function, in adults with HABP/VABP.

## MATERIALS AND METHODS

### Data sources.

Exposure–efficacy analyses were performed using data from the phase 3 ASPECT-NP clinical trial (ClinicalTrials.gov identifier: NCT02070757; protocol MK-7625A-P008) (15). The ASPECT-NP trial was conducted in accordance with principles of Good Clinical Practice and approved by the appropriate institutional review boards and regulatory agencies (15). ASPECT-NP enrolled a total of 726 adult participants with ventilated HABP/VABP who were randomized 1:1 to receive 3 g of ceftolozane/tazobactam (2 g of ceftolozane and 1 g of tazobactam) or 1 g of meropenem via intravenous infusion every 8 h for 8 to 14 days. Dosing was adjusted based on renal function, estimated using the Cockroft-Gault formula (30) (Table 1). Data from ASPECT-NP trial participants in the ceftolozane/tazobactam group of the ITT population were included if participants had both a pathogen isolated at baseline and available exposure measures from the population PK model.

### Exposure–efficacy data set and measurements.

The EEA population comprised one record per participant for each efficacy endpoint so that 28-day all-cause mortality (28 days after treatment initiation) data were captured as alive or dead and clinical cure at TOC (7 to 14 days after end of treatment) data were captured as cure or failure (includes indeterminate). Time-to-death and time-to-cure data were also included. The exposure measures for each participant and PK/PD endpoint data were combined with demographic covariates to create the data set for the exposure–response analysis of efficacy endpoints.

For exposure–efficacy analyses, the PK/PD measure evaluated for ceftolozane was %*ƒ*T>MIC for the Gram-negative pathogens isolated from baseline LRT cultures; the highest ceftolozane/tazobactam MIC values were selected in cases where participants had multiple relevant pathogens at baseline (23). For tazobactam, the PK/PD measure evaluated was the %*ƒ*T>C_T_=1 μg/mL (unpublished data) (24, 31). Individual ceftolozane/tazobactam exposure measures (i.e., %ƒT>MIC and %ƒT>C_T_) associated with the last dose for each participant were calculated based on ceftolozane and tazobactam concentration–time profile simulated in NONMEM, version 7.3.0 (ICON Development Solutions, Hanover, MD, USA), using PK population modeling and the associated individual specific parameter estimates (16). The model-predicted exposure measures associated with the last dose obtained for each participant were merged with the exposure–efficacy data set and used in the development of the exposure–efficacy models to describe the exposure–efficacy relationships.

After generation of individual exposure estimates based on the population PK models, the planned procedures for exposure–efficacy model development included graphical data exploration to assess whether there was an apparent trend between exposure measures and primary and key secondary efficacy endpoints. If a trend was identified by visual assessment, a formal logistic regression modeling analysis would be conducted to quantify the trend and to evaluate the potential impact of covariates, including age, baseline body weight, sex, baseline creatinine clearance, diagnosis at baseline, and key comorbidities (i.e., diabetes, congestive heart failure, and chronic obstructive pulmonary disease). Initial objectives for this analysis included the development of formal exposure–outcomes models for ceftolozane and tazobactam in HABP/VABP. However, the exploratory analyses demonstrated that model exploration would not provide meaningful information because the data were not normally distributed.

### PD data collection.

Efficacy measures included 28-day all-cause mortality and clinical cure at the TOC visit (7 to 14 days after the end-of-treatment visit) (15). The 28-day all-cause mortality endpoint was inclusive of all deaths that occurred on or before day 28. Clinical outcome (cure, failure, or indeterminate) was determined by the blinded investigator based on an overall evaluation of clinical status per signs, symptoms, and available laboratory data. A favorable response was clinical cure. Participants who experienced an insufficient therapeutic effect, defined as clinical worsening or lack of clinical progress, and who discontinued study treatment were defined as a clinical failure on the day they discontinued study treatment. Participants with an indeterminate clinical response, where the investigator was unable to assign an outcome of clinical cure or clinical failure because of premature study drug discontinuation, or death within 48 h of the first dose of study drug were included in the denominator when calculating clinical cure rates at the TOC visit in the ITT population.
