# Supplementary material for: Exposure–Efficacy Analyses Support Optimal Dosing Regimens of Ceftolozane/Tazobactam in Participants with Hospital-Acquired/Ventilator-Associated Bacterial Pneumonia in ASPECT-NP
Source: Antimicrob Agents Chemother. 2022 Apr 26;66(5):e01399-21. doi: 10.1128/aac.01399-21 (PMC9112930; doi:10.1128/aac.01399-21)
Supplement: Supplemental file 1 — Tables S1 and S2. Download aac.01399-21-s0001.pdf, PDF file, 0.2 MB [file aac.01399-21-s0001.pdf]

**TABLE S1** Baseline characteristics of ASPECT-NP participants by population[a]

| Characteristic | Statistic or group | EEA population dataset (*N* = 231) | ITT population (*N* = 362) |
|---|---|---|---|
| Age, yrs | Mean (SD) | 60.0 (16.4) | 60.5 (16.7) |
| | Median (min–max) | 62.0 (18–98) | 62.5 (18–98) |
| Age group, *n* (%) | <65 yrs | 131 (56.7) | 202 (55.8) |
| | ≥65 yrs | 100 (43.3) | 160 (44.2) |
| | <75 yrs | 184 (79.7) | 279 (77.1) |
| | ≥75 yrs | 47 (20.3) | 83 (22.9) |
| Body weight, kg | Mean (SD) | 81.2 (16.6) | 80.9 (17.3)[c] |
| | Median (min–max) | 80.0 (42.0–146.0) | 80.0 (34.0–150.1)[c] |
| CrCl, mL/min[b] | Mean (SD) | 108.5 (60.0) | 105.0 (59.3)[d] |
| | Median (min–max) | 97.1 (16.0–427.9) | 95.0 (16.0–427.9)[d] |
| CrCl category, *n* (%)[b] | ≥150 mL/min (ARC) | 46 (19.9) | 67 (18.5) |
| | ≥80 to <150 mL/min (normal) | 109 (47.2) | 160 (44.2) |
| | >50 to <80 mL/min (mild impairment) | 46 (19.9) | 82 (22.7) |

| Characteristic | Statistic or group | EEA population dataset (*N* = 231) | ITT population (*N* = 362) |
|---|---|---|---|
| | ≥30 to ≤50 mL/min (moderate) | 19 (8.2) | 35 (9.7) |
| | ≥15 to <30 mL/min (severe impairment) | 11 (4.8) | 17 (4.7) |
| APACHE II score | Mean (SD) | 17.4 (5.1) | 17.5 (5.2)[d] |
| | Median (min–max) | 17.0 (2.0–32.0) | 17.0 (2.0–33.0)[d] |
| SOFA score | Mean (SD) | 6.3 (2.3) | — |
| | Median (min–max) | 6.0 (0.0–15.0) | — |
| CPIS | Mean (SD) | 9.9 (1.8) | — |
| | Median (min–max) | 10.0 (5.0–13.0) | — |
| CPIS, *n* (%) | ≤6 | 14 (6.1) | 25 (6.9) |
| | 7 | 17 (7.4) | 29 (8.0) |
| | 8 | 17 (7.4) | 45 (12.4) |
| | >8 | 183 (79.2) | 263 (72.7) |
| Sex, *n* (%) | Male | 174 (75.3) | 262 (72.4) |
| | Female | 57 (24.7) | 100 (27.6) |

| Characteristic | Statistic or group | EEA population dataset (*N* = 231) | ITT population (*N* = 362) |
|---|---|---|---|
| Race, *n* (%) | Asian | 18 (7.8) | 27 (7.5) |
| | Black or African American | 6 (2.6) | 10 (2.8) |
| | White | 194 (84.0) | 301 (83.1) |
| | Native Hawaiian/Pacific Islander | 1 (0.4) | 1 (0.3) |
| | Other/not reported | 12 (5.2) | 23 (6.4) |
| Region, *n* (%) | North America | 8 (3.5) | 15 (4.1) |
| | Latin America | 13 (5.6) | 21 (5.8) |
| | Western Europe | 35 (15.2) | 53 (14.6) |
| | Eastern Europe | 147 (63.6) | 231 (63.8) |
| | Asia/Pacific | 18 (7.8) | 28 (7.7) |
| | Rest of world | 10 (4.3) | 14 (3.9) |
| Prior antibacterial therapy failure for VNP, *n* (%) | Yes | 34 (14.7) | 53 (14.6) |
| VABP diagnosis, *n* (%) | Yes | 182 (78.8) | 263 (72.7) |

| Characteristic | Statistic or group | EEA population dataset (*N* = 231) | ITT population (*N* = 362) |
|---|---|---|---|
| Diabetes, *n* (%) | Yes | 51 (22.1) | 88 (24.3) |
| Congestive heart failure, *n* (%) | Yes | 37 (16.0) | 55 (15.2) |
| Chronic obstructive pulmonary disease, *n* (%) | Yes | 15 (6.5) | 40 (11.0) |
| Bacteremic, *n* (%) | Yes | 37 (16.0) | 25 (6.9) |
| Gram-negative adjunct therapy, *n* (%) | Yes | 61 (26.4) | 103 (28.5) |
| Gram-positive adjunct therapy, *n* (%) | Yes | 224 (97.0) | 350 (96.7) |
| $PaO_2/FiO_2$ >240, *n* (%) | Yes | 56 (24.2) | 93 (25.7) |
| Empyema, *n* (%) | No | 231 (100.0) | 361 (99.7) |
| Pleural effusion, *n* (%) | No | 215 (93.1) | 323 (89.2) |
| Ventilation of ≥5 days, *n* (%) | Yes | 125 (54.1) | 182 (50.3) |
| Hospitalization of ≥5 days, *n* (%) | Yes | 179 (77.5) | 278 (76.8) |

| Characteristic | Statistic or group | EEA population dataset (*N* = 231) | ITT population (*N* = 362) |
|---|---|---|---|
| Prior antibacterial use, *n* (%) | Yes | 214 (92.6) | 318 (87.8) |
| *Enterobacterales*, *n* (%) | Yes | 170 (73.6) | 195 (73.9)[e] |
| Polymicrobial LRT pathogen, *n* (%) | Yes | 91 (39.4) | 100 (37.9)[e] |

[a]APACHE, Acute Physiology and Chronic Health Evaluation; ARC, augmented renal clearance; CPIS, Clinical Pulmonary Infection Score; CrCl, creatinine clearance; EEA, exposure–efficacy analysis; ITT, intention-to-treat; LRT, lower respiratory tract; $PaO_2$,/FiOS, ratio of partial pressure of oxygen to fractional inspired oxygen; SOFA, Sequential Organ Failure Assessment; VABP, ventilator-associated bacterial pneumonia; VNP, ventilated nosocomial pneumonia.

[b]Calculated using central laboratory data.

[c]$N = 359$.

[d]$N = 361$.

[e]$N = 264$.

**TABLE S2** Summary of 28-day all-cause mortality and clinical cure at test of cure visit for ASPECT-NP participants by baseline creatinine clearance group.[a,b]

| | **28-day all-cause mortality, *n/N* (%)** | | **Clinical cure at test of cure, *n/N* (%)** | |
|---|---|---|---|---|
| **Renal function subgroup** | **Alive** | **Death** | **Cure** | **Failure** |
| All | 195/231 (84.4) | 36/231 (15.6) | 151/231 (65.4) | 80/231 (34.6) |
| CrCl ≥15 to <30 mL/min | 8/11 (72.7) | 3/11 (27.3) | 5/11 (45.5) | 6/11 (54.5) |
| CrCl ≥30 to ≤50 mL/min | 15/19 (78.9) | 4/19 (21.1) | 8/19 (42.1) | 11/19 (57.9) |
| CrCl >50 mL/min | 172/201 (85.6) | 29/201 (14.4) | 138/201 (68.7) | 63/201 (31.3) |

[a]CrCl, creatinine clearance.

[b]Calculated using central laboratory data.
